# Evaluating ChatGPT’s Diagnostic Accuracy in Detecting Fundus Images

**DOI:** 10.7759/cureus.73660

**Published:** 2024-11-14

**Authors:** Ayushi Gupta, Hussein Al-Kazwini

**Affiliations:** 1 Ophthalmology, Royal Free Hospital, London, GBR; 2 Ophthalmology, East Kent Hospitals University National Health Service (NHS) Foundation Trust, Canterbury, GBR

**Keywords:** artificial intelligence, chatgpt, fundus photography, large language model, ophthalmology, retina

## Abstract

Introduction

Artificial intelligence is rapidly advancing in healthcare. Ophthalmology, with its reliance on imaging for diagnosis and management, has the potential to benefit from this technology. Deep learning models are currently used in image analysis in ophthalmology. ChatGPT (OpenAI, San Francisco, CA), a large language model, has recently expanded to include image analysis, creating new opportunities for diagnostic applications. While prior research shows potential in text-based diagnostics for ophthalmology, there is limited literature on AI’s diagnostic accuracy in interpreting retinal images.

Methods

We selected 12 fundus images from key diseases identified by the Royal College of Ophthalmology curricula for medical students, foundation doctors, and trainees. Each image was presented to ChatGPT 4.0 using a standardised prompt to identify the most likely diagnosis. Responses were recorded, and the model’s accuracy was assessed by comparing its diagnoses to the confirmed conditions.

Results

ChatGPT accurately diagnosed four out of 12 diseases (papilloedema, dry age-related macular degeneration (ARMD), glaucoma and vitreous haemorrhage) and provided one partially correct diagnosis (diabetic retinopathy). However, the model struggled with seven cases, including central retinal artery occlusion, central retinal vein occlusion, dry ARMD, rhegmatogenous retinal detachment, tractional retinal detachment, epiretinal membrane and macular hole.

Conclusion

ChatGPT demonstrates the potential for diagnosis of retinal conditions from fundus photography. However, it currently lacks the accuracy required for clinical application; the model often hallucinates when unsure, which has diagnostic implications. Further work is required to refine these models and expand their diagnostic potential.

## Introduction

Artificial intelligence (AI) is an emerging technology with the potential to redefine healthcare as an adjunct for diagnosis and management. There are several algorithms included under the umbrella of AI, including deep learning (DL), machine learning (ML) and large language models (LLM) [1]. Ophthalmology lends itself to the incorporation of AI due to its reliance on imaging, including fundus photography and optical coherence tomography (OCT). Currently, AI has been successfully implemented in diabetic retinopathy screening, in which there has been automation of diagnostic steps through the use of approved DL AI tools, reducing the burden on graders [2].

ChatGPT, developed by OpenAI (San Francisco, CA), is an example of a widely available LLM trained on high volumes of data and initially designed for text-based applications. More recent ChatGPT LLM models incorporate the analysis of images, presenting a new opportunity for diagnostic tools and screening programmes [3]. Several studies have noted the ability of ChatGPT and competitor models such as Google Gemini/Bard (Google AI, San Francisco, CA) or Microsoft Bing (Microsoft® Corp., Redmond, WA) to provide diagnostic insights for text-based cases across a range of ophthalmological subspecialties including neuro-ophthalmology, refractive surgery and retina [4-7]. These studies identify that while there is some difference in performance between models, memory features allow for adaptive learning and continuous improvement in accuracy [7]. However, there are ongoing issues with a lack of completeness or hallucination, a phenomenon where AI generates misleading answers when models cannot predict the correct answer [8].

Despite these advances, there is a lack of literature assessing the diagnostic accuracy of LLM AI in image interpretation. In a speciality that relies heavily on imaging adjuncts for diagnosis, this represents a gap in the literature [5]. This paper aims to evaluate ChatGPT’s diagnostic accuracy in detecting retinal disease from fundus photographs to discuss implications for future diagnostics.

## Materials and methods

This study identified fundus photographs of 12 important retinal diseases highlighted by the Royal College of Ophthalmologists (RCOphth) curricula for medical students, foundation year doctors and ophthalmology trainees, shown in the figure in the Results section [9,10]. The following conditions were highlighted from the RCOphth curriculum for medical students and foundation doctors: central retinal artery occlusion (CRAO), central retinal vein occlusion (CRVO), papilloedema, wet age-related macular degeneration (ARMD), dry ARMD, proliferative diabetic retinopathy (PDR), rhegmatogenous retinal detachment and vitreous haemorrhage [9]. Meanwhile, the following conditions were highlighted by the RCOphth for ophthalmology trainees: optic disc atrophy, tractional retinal detachment, epiretinal membrane and macular hole [10]. 

One open-access fundus image was obtained for each of the above conditions under the Creative Commons Licence. The photographs were selected based on image clarity, both in terms of image resolution and single pathology and provision of a stated diagnosis by a healthcare professional. The diagnosis was corroborated by two medical professionals. Following this, we devised a prompt for the AI model: ‘Based solely on the following fundus image of the retina, what is the most likely diagnosis?’. This question was designed to return an accurate, concise diagnosis. 

The respective images for each retinal condition and the prompt were posted together in ChatGPT, and the response for each is recorded in the table in the Results section. ChatGPT version 4.0 was used as an example of a widely available LLM with image analysis properties in this paper. The memory function of ChatGPT was turned off, and a new thread was opened each time the question was posed to mitigate against adaptive learning. Each image was presented twice to ensure the reproducibility of answers. In instances where multiple diagnoses were returned, the first diagnosis was selected.

## Results

**Figure 1:**
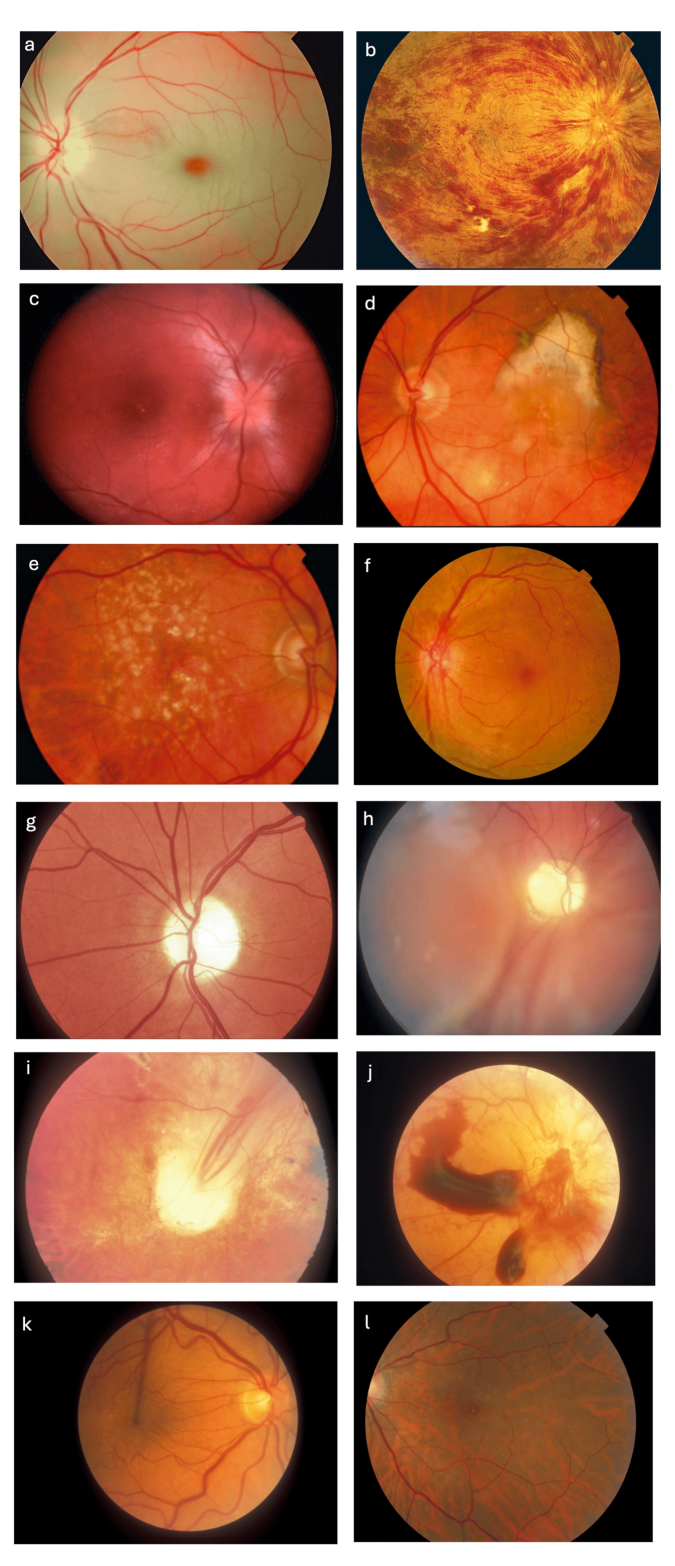
Fundus Images Fundus images were presented to ChatGPT with the following diagnoses: a) CRAO, b) CRVO, c) papilloedema, d) wet ARMD, e) dry ARMD, f) PDR, g) glaucoma, h) rhegmatogenous retinal detachment, i) tractional retinal detachment, j) PDR with vitreous haemorrhage, k) epiretinal membrane and l) macular hole. ARMD, age-related macular degeneration; CRAO, central retinal artery occlusion; CRVO, central retinal vein occlusion; PDR, proliferative diabetic retinopathy

The fundus images used are shown in Figure 1, and the results are summarised in Table 1.

**Table 1:** Fundus Images and Diagnoses The actual ChatGPT diagnoses for the corresponding fundus images are in Figure 1. The full ChatGPT explanations for diagnoses are included. ARMD, age-related macular degeneration; CRAO, central retinal artery occlusion; CRVO, central retinal vein occlusion; PDR, proliferative diabetic retinopathy; RCOphth, Royal College of Ophthalmologists

Fundus Image	Actual Diagnosis	ChatGPT Diagnosis	ChatGPT Full Response	Curriculum That Fundus Image Is Relevant to [9,10]
a	CRAO	Macular Degeneration	Possible considerations based on this image could include the following: (1) Macular degeneration: If there is a significant disruption around the macula. (2) Diabetic retinopathy: If there are any haemorrhages, exudates, or vascular changes. (3) Optic disc oedema: If the optic disc appears swollen or has unclear margins. (4) Central serous chorioretinopathy (CSCR): If there is a central area of serous fluid causing elevation.	RCOphth Curriculum for Undergraduates and Foundation Doctors
b	CRVO	Hypertensive Retinopathy	The most likely diagnosis is hypertensive retinopathy. The features that support this diagnosis include the following: (1) Arteriolar narrowing and a "copper wire" or "silver wire" appearance of the vessels. (2) Cotton wool spots: These are areas of microinfarction of the nerve fibre layer. (3) Hard exudates: Yellowish lipid deposits, which may be seen in a star-shaped pattern around the macula. (4) Haemorrhages: Various retinal haemorrhages, which could be flame-shaped or dot-and-blot.	RCOphth Curriculum for Undergraduates and Foundation Doctors
c	Papilloedema	Papilloedema	The most likely diagnosis is papilledema. The key findings that suggest this include the following: (1) Swelling of the optic disc: The margins of the optic disc appear blurred, which is a characteristic sign of papilledema. (2) Engorgement of blood vessels: The vessels near the optic disc seem congested. (3) Hyperaemia: The optic disc appears more reddish due to increased blood flow and congestion.	RCOphth Curriculum for Undergraduates and Foundation Doctors
d	Wet ARMD	CSCR	Based on the fundus image you provided, which shows a pale, yellowish, elevated lesion with irregular borders and surrounding chorioretinal atrophy, the most likely diagnosis is CSCR. The pale, elevated area, often seen with some yellowish pigment changes, can suggest serous retinal detachment or pigment epithelial detachment commonly associated with CSCR. The lesion appears to be subretinal, and these features are typically found in patients with CSCR, particularly in chronic cases.	RCOphth Curriculum for Undergraduates and Foundation Doctors
e	Dry ARMD	Dry ARMD	The fundus image shows a retina with multiple yellowish deposits, particularly concentrated in the macular area. These deposits, known as drusen, are characteristic of ARMD, specifically the "dry" or non-exudative type. ARMD is a common cause of vision loss in older adults, and the presence of drusen is a hallmark feature of the condition, particularly in its early and intermediate stages.	RCOphth Curriculum for Undergraduates and Foundation Doctors
f	PDR	Non-proliferative Diabetic Retinopathy (NPDR)	Based on the appearance of the retina in the provided fundus image, the most likely diagnosis is diabetic retinopathy (likely NPDR). (1) Dot and blot haemorrhages: The image shows dark spots consistent with intraretinal haemorrhages, a hallmark of diabetic retinopathy. (2) Microaneurysms: The small, round, red dots seen in the image may represent microaneurysms, which are often the earliest visible sign of diabetic retinopathy. (3) Hard exudates: The yellowish spots are likely hard exudates, indicating leakage of lipid and proteinaceous material from blood vessels, another common feature in diabetic retinopathy. (4) Vascular abnormalities: Tortuous and dilated blood vessels can also be seen, consistent with changes in the vasculature due to diabetes.	RCOphth Curriculum for Undergraduates and Foundation Doctors
g	Glaucoma	Glaucoma	Optic disc cupping, suggestive of glaucoma. (1) Increased optic disc cupping: The optic disc shows a large, pale central area (the cup), indicating an increased cup-to-disc ratio, which is characteristic of glaucomatous optic neuropathy. In glaucoma, the optic nerve head undergoes atrophy, resulting in a deeper and larger cup. (2) Pallor of the optic disc: The pale appearance of the optic disc centre suggests atrophy, often associated with glaucoma. (3) Thin neuroretinal rim: There appears to be a thinning of the neuroretinal rim, another common finding in glaucoma.	Trainees Handbook
h	Rhegmatogenous Retinal Detachment	Papilloedema	This fundus image shows optic disc swelling with blurred disc margins, which is consistent with papilledema. Papilledema is swelling of the optic disc due to increased intracranial pressure. The optic disc appears elevated, and the normal sharp borders are obscured, which are hallmark features of this condition.	RCOphth Curriculum for Undergraduates and Foundation Doctors
i	Tractional Retinal Detachment	Optic Atrophy	This fundus image shows optic atrophy, characterized by a pale optic disc with well-defined margins and loss of small blood vessels on the disc surface, known as "disc cupping." Optic atrophy can result from damage to the optic nerve due to various causes, such as glaucoma, previous optic neuritis, ischemic optic neuropathy, or compressive lesions on the optic nerve.	Trainees Handbook
j	PDR With a Vitreous Haemorrhage	PDR With a Vitreous Hemorrhage	Based on the third fundus image, the most likely diagnosis is PDR with a vitreous haemorrhage. (1) Vitreous haemorrhage: The large, dark, and irregular blood collection visible suggests bleeding into the vitreous cavity, a common complication of advanced diabetic retinopathy. (2) Neovascularization: The presence of new, abnormal blood vessels (often fragile and prone to bleeding) is typical in proliferative diabetic retinopathy. (3) Fibrous proliferation: The image shows signs of fibrous tissue that may accompany neovascularization, contributing to the risk of tractional retinal detachment.	RCOphth Curriculum for Undergraduates and Foundation Doctors
k	Epiretinal Membrane	Branch Retinal Vein Occlusion (BRVO)	Based solely on the appearance of the second fundus image, the most likely diagnosis appears to be BRVO. (1) Darkened or shadowed areas: There appears to be a segment of the retina with darker vessels and a possible disruption in the vascular structure, suggesting impaired blood flow. (2) Venous dilation and tortuosity: Affected areas often display these features due to the blockage in venous drainage. (3) Segmental distribution: BRVO typically affects a quadrant or a specific section of the retina, which aligns with the visible pattern in this image.	Trainees Handbook
l	Macular Hole	Mild NPDR	Based on the provided fundus image, the most likely diagnosis appears to be mild NPDR. This is suggested if there are features such as microaneurysms, slight retinal haemorrhages, or subtle changes in the retinal vasculature, which can sometimes be challenging to discern in NPDR. From a high-level view, here are a few other potential retinal conditions that can be considered based on common appearances: (1) Diabetic retinopathy: This condition often shows features such as microaneurysms, haemorrhages, exudates, or neovascularization. However, the presence of these changes is not clear from this image. (2) Hypertensive retinopathy: Signs may include arteriolar narrowing, changes in the arteriovenous crossing, or flame-shaped haemorrhages. The image does not appear to show these obvious signs. (3) ARMD: Usually characterized by drusen (yellowish deposits) or signs of neovascularization around the macula. This does not seem to be apparent. (4) CSCR: May present with a localized detachment of the retina, which could be subtle but does not seem to show clearly in this case. (5) Retinal detachment or tear: This would typically show areas of elevation or disruption, which does not seem visible here.	Trainees Handbook

ChatGPT was able to provide a single answer for the most likely diagnosis for 11 of the 12 photographs, providing an explanation of the key features leading to the answer (Table 1). In one case, CRAO, the model was unable to choose a single diagnosis (Table 1). Note that in the macular hole case, the model elaborated with other differentials (Table 1). In all cases, ChatGPT returned the same answer on both attempts; therefore, only the first ChatGPT response is shown in Table 1. 

ChatGPT provided correct answers to four fundus photographs: papilloedema (Figure 1c), dry ARMD (Figure 1e), glaucoma (Figure 1g) and vitreous haemorrhage secondary to PDR (Figure 1j). The model was partially correct in one scenario, identifying diabetic retinopathy but classifying the disease as NPDR rather than PDR (Figure 1f). ChatGPT was incorrect in seven fundus photographs: CRAO (Figure 1a), CRVO (Figure 1b), wet ARMD (Figure 1d), rhegmatogenous and tractional retinal detachments (Figure 1h, Figure 1i), epiretinal membrane (Figure 1k) and macular hole (Figure 1l). 

For the RCOphth Curriculum for Undergraduates and Foundation Doctors, ChatGPT was able to correctly identify three out of eight fundus photographs, and one out of these eight was partially correct (Table 1). For the Trainees Handbook Curriculum, ChatGPT was able to correctly identify one out of four fundus photographs (Table 1).

## Discussion

Our study suggests that while ChatGPT, an LLM, can provide correct diagnoses for important retinal conditions from fundus photographs, there are ongoing reliability and accuracy issues with an inability to accurately diagnose seven of the 12 fundus photographs presented. ChatGPT provided a partially correct diagnosis in the diabetic retinopathy case, suggesting that while it can identify patterns of disease, it may require additional support to recognise nuances of the disease, particularly in cases where the model has not been trained [11]. The poor diagnostic accuracy and specificity highlight the limitations of LLMs in ophthalmological image interpretation. Despite advances in DL and ML, LLMs are yet to be optimised for image processing and also rely on pre-trained language-based data [12,13]. Therefore, in the context of clinical image interpretation, DL and ML models remain superior [12]. 

In cases where the model was uncertain of the answer, as seen in CRAO and, to some extent, the macular hole, ChatGPT would provide a number of diagnoses. This reduces the reliability and accuracy of answers. Furthermore, the model appeared to hallucinate in situations when unsure, which is a phenomenon observed with ChatGPT [14,15]. Hallucinations describe fabrications from the AI server to justify its answer. For example, in the epiretinal membrane example above, the model described features of venous dilation that are not present to justify its answer of branch retinal vein occlusion (BRVO) (Figure 1, Table 1). Hallucinations are also a key limitation of text-based LLMs, and concerns around misleading information pose a limitation for the use of AI as a diagnostic aid [8]. 

The difference between ChatGPT's promising text-based diagnostic capability described in the literature [4-7] and its current limitations in image analysis seen in this study highlights the need for algorithms designed for medical imaging, as seen with existing DL models successfully used in diabetic retinopathy screening. In clinical practice, images are used as an aid by healthcare professionals following extensive history and examination; without this additional information, ChatGPT is at a disadvantage. On the other hand, it can be argued that one of the key developments of AI in ophthalmology is in automated grading or screening of images. In such cases, models must be able to identify pathology in the absence of patient history. 

This study is limited in its small sample size and use of a single model. Future work should collate several fundus photographs from each condition, branch into other imaging modalities, and consider other LLMs. This will enable further developments and training of LLM AI tools for image analysis and diagnostic support in ophthalmology.

## Conclusions

This study identifies that while there is promise in using LLMs, such as ChatGPT, as diagnostic adjuncts in fundus image interpretation in ophthalmology, there are still limitations with accuracy. In its current form, the model is unable to reliably diagnose key fundal pathology and hallucinates when unsure. As LLMs evolve, additional algorithms should be developed to enhance accuracy. This approach could improve diagnostic accuracy and potentially reduce the workload on healthcare providers, particularly in ophthalmology, where imaging is central to patient management.
